# Schistosomiasis manifesting as a colon polyp: a case report

**DOI:** 10.1186/1752-1947-8-331

**Published:** 2014-10-08

**Authors:** Iyad Issa, Mona Osman, Georges Aftimos

**Affiliations:** 1 Department of Gastroenterology and Hepatology, Rafic Hariri University Hospital (RHUH), Beirut, Lebanon; 2 Institut National de Pathologie (INP), Baabda, Lebanon; 3 Specialty Clinics Center 4B, Hamra, 2034-7304 Beirut, Lebanon

**Keywords:** Colon polyp, *Mansoni*, Parasite, *Schistosoma*

## Abstract

**Introduction:**

Schistosomiasis is a rare disease with a common intestinal involvement. However, colon polyps associated with *Schistosoma* in the absence of inflammation have rarely been reported, especially in young people; this is the first case with the following presentation.

**Case presentation:**

We describe the case of a 20-year-old Ethiopian woman living in Lebanon who presented with nonspecific abdominal symptoms. Her biochemical profile was normal in addition to the results of her stool and urine tests. A colonoscopy showed normal colonic mucosa but surprisingly a large pedunculated polyp was found in her ascending colon. Pathology revealed a hamartomatous polyp but it was full of partially calcified parasitic eggs of *Schistosoma mansoni* compatible with chronic schistosomiasis.

**Conclusions:**

She was treated with two doses of praziquantel and showed immediate marked clinical improvement. This unusual case will give us the opportunity to discuss schistosomiasis, its occurrence in colon polyps, clinical significance and the various means of management.

## Introduction

Schistosomiasis is a trematodes infection, sometimes referred to as “bilharziasis”. It is a serious endemic disease in the tropics and subtropics. More than 200 million people are affected in the world and several other millions are exposed to the infection [1,2]. Intestinal involvement is common. Colon polyps associated with the disease although previously reported are quite rare. We report a case of a colonic polyp associated with chronic schistosomiasis.

## Case presentation

**Figure 1:**
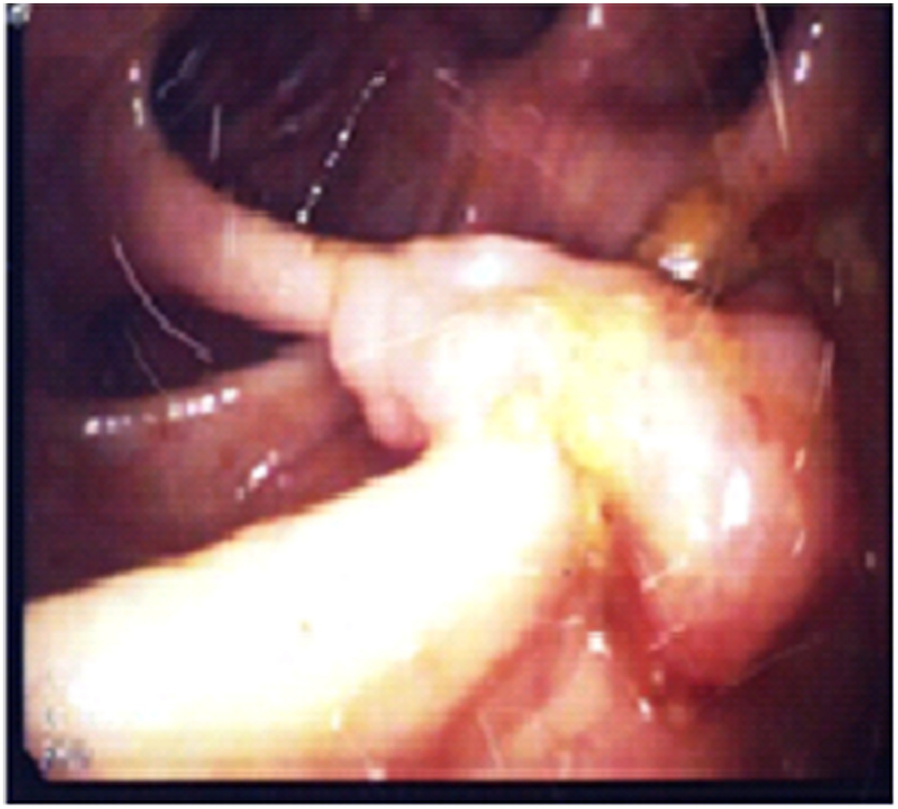
**Endoscopic appearance of the polyp.** The colonoscopy showed a large narrow-based pedunculated polyp in the ascending colon.

**Figure 2:**
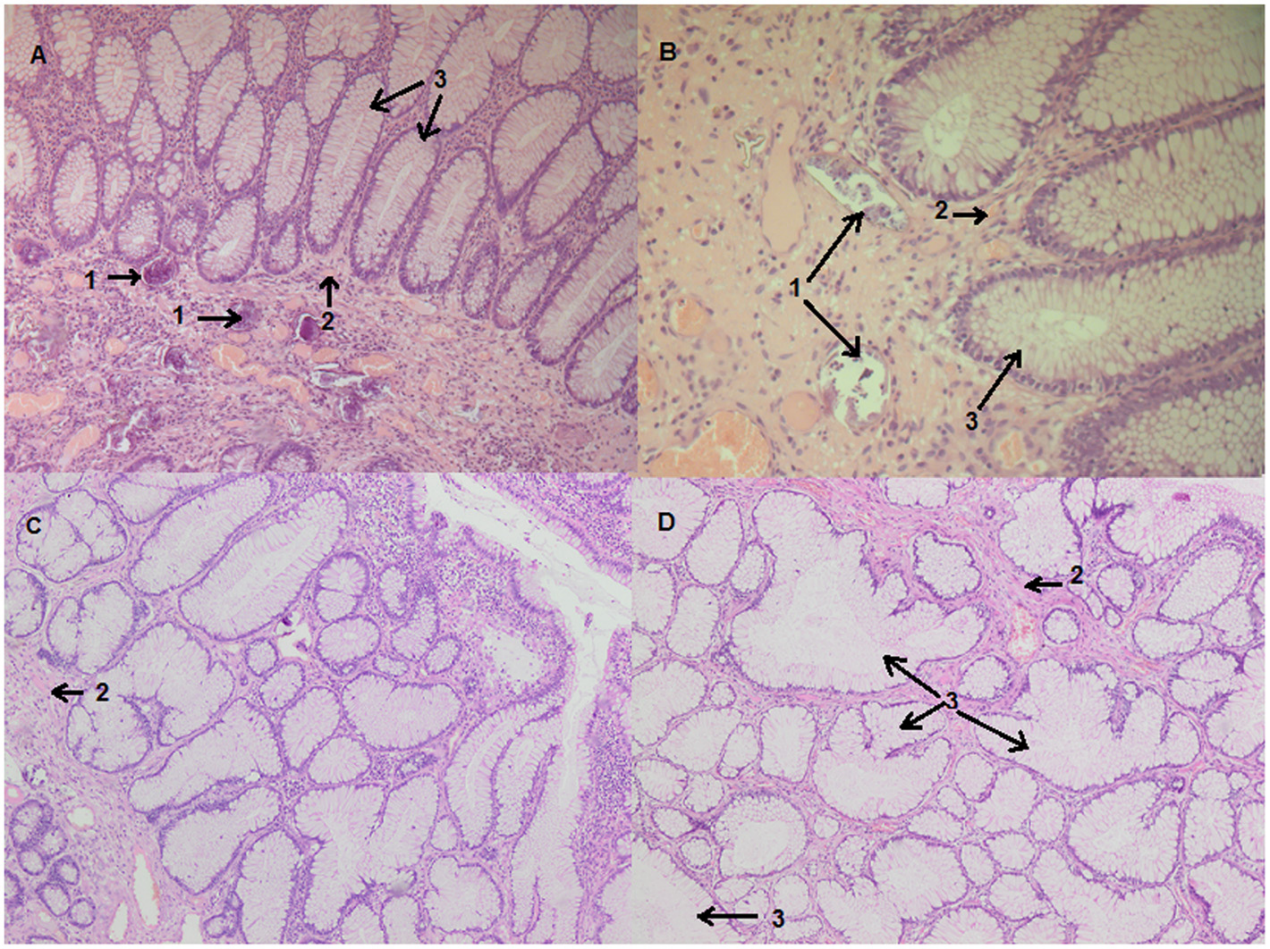
**Hematoxylin and eosin staining of the pathology slide.** The submitted sample is a polypoid formation measuring 1.8×0.8×0.6cm with a short pedicle. The performed cuts **(A, B, C, D)** are stained with hematoxylin and eosin; magnification ×25. They show a benign hamartomatous polyp, containing multiple partially calcified schistosoma eggs (1); the stroma contains smooth muscular fibers (2) intermingled with the branching and variably oriented glands (3); these glands are lined by intestinal-type epithelium with predominant goblet cells and few paneth cells at the bottom of these glands.

**Figure 3:**
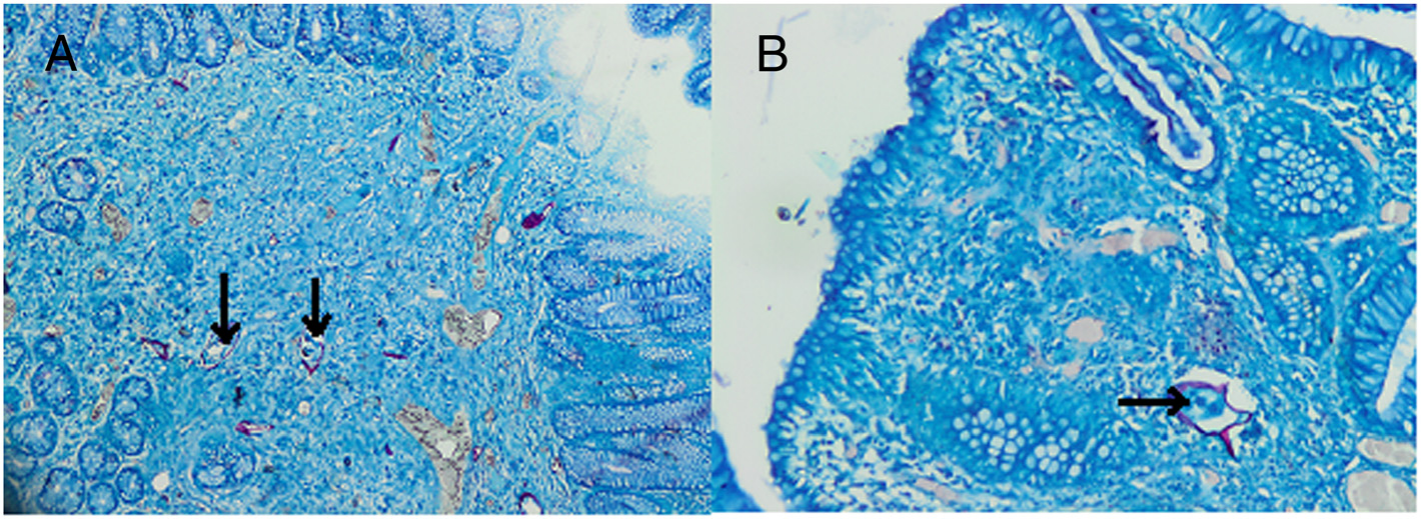
**Ziehl–Neelsen staining of the pathology slide.** These two pictures demonstrate the *Schistosoma* eggs after Ziehl–Neelsen coloration. Magnification **A** ×40, **B** ×200. *Schistosoma mansoni* eggs are elongate and possess a lateral spine. The shell is acid-fast when stained with Ziehl–Neelsen stain (arrows).

A 20-year-old Ethiopian woman who has lived in Lebanon for the past 3 years presented to our hospital in Beirut, Lebanon, for diffuse colicky abdominal pain and bloating of a couple of years’ duration. This was exacerbated by food intake and relieved by defecation and flatus emission. Symptoms were associated with several bouts of nausea and vomiting. She also reported constipation, with several episodes of mucous in stool, and one episode of moderate amount of fresh blood per rectum. She denied fever, anorexia or weight loss. Her blood profile was normal except for mild anemia (Table 1); the results of her urine and stools tests were also normal (Table 2). A previously done ultrasound of her abdomen had revealed no abnormalities. A colonoscopy was performed and showed normal colonic mucosa but surprisingly a large (2cm) pedunculated polyp was noted in her ascending colon (Figure 1). It was removed by snare under bipolar cautery and sent to the pathologist. The pathologist was puzzled to report a hamartomatous polyp full of partially calcified parasitic *Schistosoma mansoni* eggs (Figures 2 and 3). Random colonic biopsies were taken and showed completely normal mucosa.

**Table 1:** Laboratory profile of the patient

**Complete blood count with differential**	**Results**	**Normal range**
White blood cell	5.9	4–11 10^9^/L
Neutrophil	60.7	40–65%
Lymphocyte	26.4	20–45%
Monocyte	7.5	2–10%
Eosinophil	4.9	1–6%
Basophil	0.5	0–1%
Hemoglobin	**11.7**	12–17g/dL
Hematocrit	**34.5**	38–51g/dL
Mean corpuscular volume	85	80–94fL
Mean corpuscular hemoglobin	28.9	27–31pg
Random distribution of red cell width	15	11.5–13.4
Platelets	194	150–400 10^9^/L
Aspartate aminotransferase	25.17	0–50IU/L
Alanine aminotransferase	12.75	0–31IU/L
Gamma-glutamyltransferase	10.78	7–64IU/L
Alkaline phosphatase	64.19	35–105IU/L
Bilirubin, Total	0.77	0.1–1.2mg/dL
Bilirubin, Direct	0.23	0–0.3mg/dL
Bilirubin, Indirect	0.54	0–1mg/dL

**Table 2:** Urine and stool tests results of the patient

**Urine analysis**	**Results**	**Normal range**
Nitrite	Negative	Negative
White blood cell	0–2	0–2/high-power field
Red blood cell	1–3	1–3/high-power field
Bacteria	Absent	Absent
Parasites	Absent	Absent
**Stool analysis**		
Occult blood	Negative	Negative
White blood cell	Negative	Negative
Fat	Negative	Negative
Bacteria	Normal flora	Normal flora
Parasites	Absent	Absent

## Discussion

Schistosomiasis if not diagnosed and treated early, might lead to complications such as chronic intestinal schistosomiasis and hepatosplenic schistosomiasis. Both have limited mortality but their morbidity is widespread [1]. Two major species of schistosomes commonly produce gastrointestinal diseases: *S. mansoni* and *Schistosoma japonicum*. In endemic areas, the infection is usually acquired in childhood [3]. The prevalence and intensity of infection rises with age and peaks at approximately 15 to 20 years. In older adults, the prevalence of infection does not change significantly, but the intensity (parasite burden) decreases dramatically [4]. Only 5 to 10% of patients with schistosomiasis will be heavily infected, and the remainder will have mild to moderate disease [5]. Therefore, most patients are asymptomatic, but when symptoms are present, they are localized either to specific organs or generalized [6]. They can be early or late depending on the stage of infection [7]. Acute symptoms may present as a swimmer’s itch (a localized dermatitis that can result in a pruritic papular or urticarial rash at the site of larval entry) or Katayama fever (a systemic hypersensitivity reaction) [3,8]. Chronic complications may be intestinal, hepatosplenic, urinary, neurological and/or pulmonary.

The symptoms of colonic schistosomiasis are nonspecific, and may mimic other gastrointestinal problems, like nonspecific abdominal pain, diarrhea, constipation, alternating bowel habits or bleeding per rectum [1]. Severe pathologies have been reported with schistosomiasis such as hemorrhagic diarrhea, obstruction secondary to an inflammatory mass, acute appendicitis, intestinal intussusception arising from a mucocele of the appendix, and ischemic colitis [9-13]. Few cases of colon cancer associated with *S. Japonica* are reported; nonetheless, the mechanism of the parasitic infection leading to carcinoma is unclear and the association is not well established [1,14-16].

Means of diagnosis vary with the period from time of infection and the endemicity of the region. Acute illness is often associated with eosinophilia in the blood and tissues; with chronic disease, peripheral eosinophilia maybe minimal or absent, while tissue eosinophilia persists. For a patient coming from an endemic country with a suspected light infection (low worm burden), the reasonable approach would be to initially use a high sensitivity diagnostic test such as a serum antibody titer. Alternatively, polymerase chain reaction assays on patients’ urine samples have shown sensitivity as high as 94.4% and were 99.9% specific for the diagnosis of schistosomiasis [17]. Other possibilities include serologic tests for the detection of one of the two gut-associated parasite proteins: the circulating anodic antigen (CAA) and the circulating cathodic antigen (CCA) [18]. Another means involves the demonstration of parasite eggs in the stool by microscopic examination (Kato-Katz smear).

Diagnostic modalities take a prime importance in endemic areas where the search for the optimal test is still ongoing. Both CCA test scores and fecal egg count carry low sensitivity in the setting of low infection. In addition, they tend to vary in infected patients on a day-to-day basis and constitute a suboptimal means for screening [19]. Therefore, researchers often stress the need for more than one sample whether urine or stool [19]. In compliance with the resolution of the World Health Assembly passed in 2012 aimed at eliminating schistosomiasis, several CCA urine cassettes have been forwarded [20] in addition to new antibody-based serum tests [21]. The cornerstone in the confirmation of the diagnosis is still considered the serologic antibody test, which although more expensive carries higher sensitivity and specificity (97% and 91% respectively) [22]. Endoscopic findings contribute to the diagnosis but they are nonspecific. However, in combination with its clinicopathologic characteristics, the diagnosis of intestinal schistosomiasis can be established [1,14]. Rectal biopsy was used for decades as a simple effective diagnostic technique at the individual clinical level; it provides an efficient way of visualizing eggs. Biopsies may be taken from the rectal valve via a biopsy forceps through the colonoscope [23]. In our case, no parasites or eggs were detected in the biopsies of the mucosa suggesting a low worm burden. Intestinal involvement in *S. mansoni* infection is usually confined to the ileum and colon, but duodenal involvement has also been reported [24]. In the early stage of the disease, the colonoscopy can range from normal mucosa to edematous, congestive mucosa, petechial hemorrhage or even frank ulcerations. In the late stage, it may show thickened bowel wall, elevated yellow nodules, polyps and/or intestinal stricture [14]. Pathology differs between acute and chronic disease. In acute disease, the *Schistosoma* ova are deposited in the lamina propria with infiltration of eosinophilic and neutrophilic granulocytes. In chronic disease, the *Schistosoma* ova are calcified and deposited with infiltration of lymphocytes and plasma cells in the submucosa and the lamina propria, with giant cell reaction. Atrophy of intestinal villi, reduction of intestinal glands and different degrees of fibrosis were also observed in chronic schistosomal colitis patients [14]. Colon polyps associated with *Schistosoma* have only been reported on few occasions. They arise due to granulomatous inflammation surrounding eggs deposited in the bowel wall. A granulomatous colitis with pseudo polyp has been reported [25]. It can present sometimes as a mass adherent to the abdominal wall or adjacent organs. In one study done at the Armed Forces Hospital, Riyadh, they evaluated 216 patients with intestinal schistosomiasis, only eight had intestinal polyps, three of them were rectal and five colonic [1]. However, they did not mention whether those patients had inflammation or not but the eight polyps showed schistosomal ova. In addition, the correlation between the endoscopic findings and the prevalence of symptoms in those patients was not well defined.

Treatment should be offered to all patients with evidence of infection regardless of presence or absence symptoms. The treatment of choice for all schistosome species is praziquantel. The recommended doses are 40mg/kg in one or two doses for *Schistosoma haematobium, S. mansoni*, and *Schistosoma intercalatum* infections and 60mg/kg in two or three doses at least 3 hours apart for *S. japonicum* and *Schistosoma mekongi*. The cure rate with praziquantel is more than 85%; if failure, then retreatment results in success in more than 80% of cases. The main therapeutic aim of praziquantel is worm load reduction, which is usually enough to relieve symptoms. If eradication is warranted then other therapeutic options include repeated treatments with praziquantel. If this fails, oxamniquine alone or in combination with praziquantel and trioxolane can be used as second line therapy [26]. However, oxamniquine is comparatively expensive and one study suggested that combination treatment did not yield superior results to either drug alone [27]. The follow up and the efficacy of treatment can be assessed by the loss of circulating antigens as this usually indicates cure; although, sometimes light infection can be missed because the unreliable correlation between egg excretion and worm burden can be misleading. Studies have shown that after successful therapy, antigen tests become negative as early as 5- to 10-days post-treatment [28]. The follow up by stool analysis (in case positive before treatment) or by colon biopsy (in case of mucosal involvement before treatment) should be delayed for at least 6 weeks post-treatment to assess cure in endemic areas. In non-endemic areas where the risk of reinfection is low, re-examination is usually recommended at 3 and 6 months [8]. In case of colon polyps, there is no available data in the literature regarding the endoscopic follow up and monitoring for recurrence.

In our case, the patient is from an endemic area (Ethiopia). The presence of a colon polyp and the calcified eggs of *S. mansoni* inside the polyp in the absence of any other explanation for the patient’s symptoms (despite the absence of inflammation) were considered diagnostic of chronic intestinal schistosomiasis. The absence of parasites in stools does not rule out the schistosomiasis. The serological tests including the CAA and the CCA are not usually needed to confirm the diagnosis; thus, they were not done in this case. Our patient was treated with praziquantel (Biltricide®) 600mg eight tabs divided into two doses 6 hours apart. She showed immediate marked clinical improvement and most of her symptoms disappeared within 2 days. She presented 6 weeks later for follow up and reported no abdominal symptoms. An additional encounter 6 months later revealed a normal-looking patient with no systemic symptoms and a normal physical examination, further investigation was not deemed necessary at this point. After this date she was lost to follow up.

## Conclusions

Schistosomiasis is a rare disease with a rather common intestinal involvement. However, colon polyps associated with *Schistosoma* are rarely reported, especially in young people. Clinical symptoms and laboratory tests are nonspecific as well as the endoscopic features, but in combination with the histopathological findings, the correct diagnosis can be established with an adequate margin of accuracy. The treatment of choice of all species of *Schistosoma* is praziquantel with a high cure rate. Disease manifesting solely as a colon polyp was not previously reported in the literature and therefore there are a lack of data on the ways of follow up.

## Consent

Written informed consent was obtained from the patient for publication of this case report and any accompanying images. A copy of the written consent is available for review by the Editor-in-Chief of this journal.

## Abbreviations

CAA: The circulating anodic antigen; CCA: The circulating cathodic antigen.

## Competing interests

The authors report no conflict of interest in the writing of this manuscript.

## Authors’ contributions

MO wrote the initial draft of the manuscript and did the literature review, GA stained and colored the slides and penned down the pathologic description, II edited and adjusted the initial draft and performed the English language polishing. All authors read and approved the final manuscript.
